# Clinical emergence of inducible macrolide resistance mediated by acquired *erm*(41) C28T mutation in *Mycobacterium abscessus*

**DOI:** 10.1128/aac.01153-25

**Published:** 2025-11-06

**Authors:** Guiqing He, Shushu Zeng, Lilla Terezia Banki, Wenzhen Zhou, Qingyong Zheng, Quelu Chen, Xiaoyan Yang, Jie Luo, Jiajun Pan, Tao Luo

**Affiliations:** 1 Department of Infectious Diseases, Wenzhou Central Hospital, The Dingli Clinical College of Wenzhou Medical University 223520, Wenzhou, China; 2 Laboratory of Infectious Diseases, Wenzhou Central Hospital, The Dingli Clinical College of Wenzhou Medical University 223520, Wenzhou, China; 3 Department of Pathogen Biology, West China School of Basic Medical Sciences & Forensic Medicine, Sichuan University 12530, Chengdu, China; 4 Department of Radiology, Wenzhou Central Hospital, The Dingli Clinical College of Wenzhou Medical University 223520, Wenzhou, China; City St George's, University of London, London, United Kingdom

**Keywords:** *Mycobacterium abscessus*, macrolide resistance, adaptive mutations, ribosomal methyltransferase

## Abstract

Inducible macrolide resistance mediated by the *erm*(41) T28 allele is a well-established intrinsic mechanism in *Mycobacterium abscessus*. Here, we report a case of *M. abscessus* pulmonary disease in which an initial *erm*(41) C28 isolate developed inducible resistance through a *de novo* C28T point mutation during prolonged therapy. To our knowledge, this case provides the first clinical evidence that *erm*(41)-mediated inducible resistance can arise *in vivo* under antimicrobial selective pressure.

## INTRODUCTION

*Mycobacterium abscessus*, one of the most drug-resistant non-tuberculous mycobacteria (NTM), is increasingly encountered in patients with chronic lung diseases such as bronchiectasis and cystic fibrosis (1, 2). Due to its intrinsic resistance to most first-line anti-tuberculosis agents and numerous other antimicrobials, managing *M. abscessus* infections poses a major therapeutic challenge (3, 4). Macrolides, such as azithromycin and clarithromycin, remain the cornerstone of therapy and are administered in combination regimens with agents including amikacin, tigecycline, cefoxitin, and imipenem (5–8). Despite intensive multidrug therapy, treatment success rates remain low (30–50%), with frequent relapse (3, 9).

*M. abscessus* exhibits macrolide resistance via two primary mechanisms: acquired resistance via mutations in the *rrl* encoding 23S rRNA (10–12), and intrinsic resistance mediated by the *erm*(41) (13). The *erm*(41) encodes a ribosomal methyltransferase that methylates adenine at position 2058 (A2058) of 23S rRNA, thereby reducing the binding affinity of macrolides. Since *erm*(41) is expressed at low basal levels but is inducible upon macrolide exposure, resistance typically manifests after 1–2 weeks of therapy, exhibiting inducible resistance (13). Clinical *M. abscessus* isolates vary in their *erm*(41) sequence, particularly at nucleotide position 28, resulting in two major genotypes: T28 and C28 (14). The T28 variant encodes a functional methyltransferase, conferring inducible resistance, whereas the C28 variant encodes a non-functional enzyme, rendering isolates susceptible to macrolides and generally associated with higher treatment success (11, 15). As both variants are considered naturally occurring polymorphisms (15), inducible resistance from the T28 variant is traditionally regarded as intrinsic. In this study, we describe a case of *M. abscessus* pulmonary disease in which an initial *erm*(41) C28 strain acquired a *de novo* C28T mutation during prolonged antibiotic exposure, resulting in phenotypic conversion to inducible macrolide resistance.

**Fig 1:**
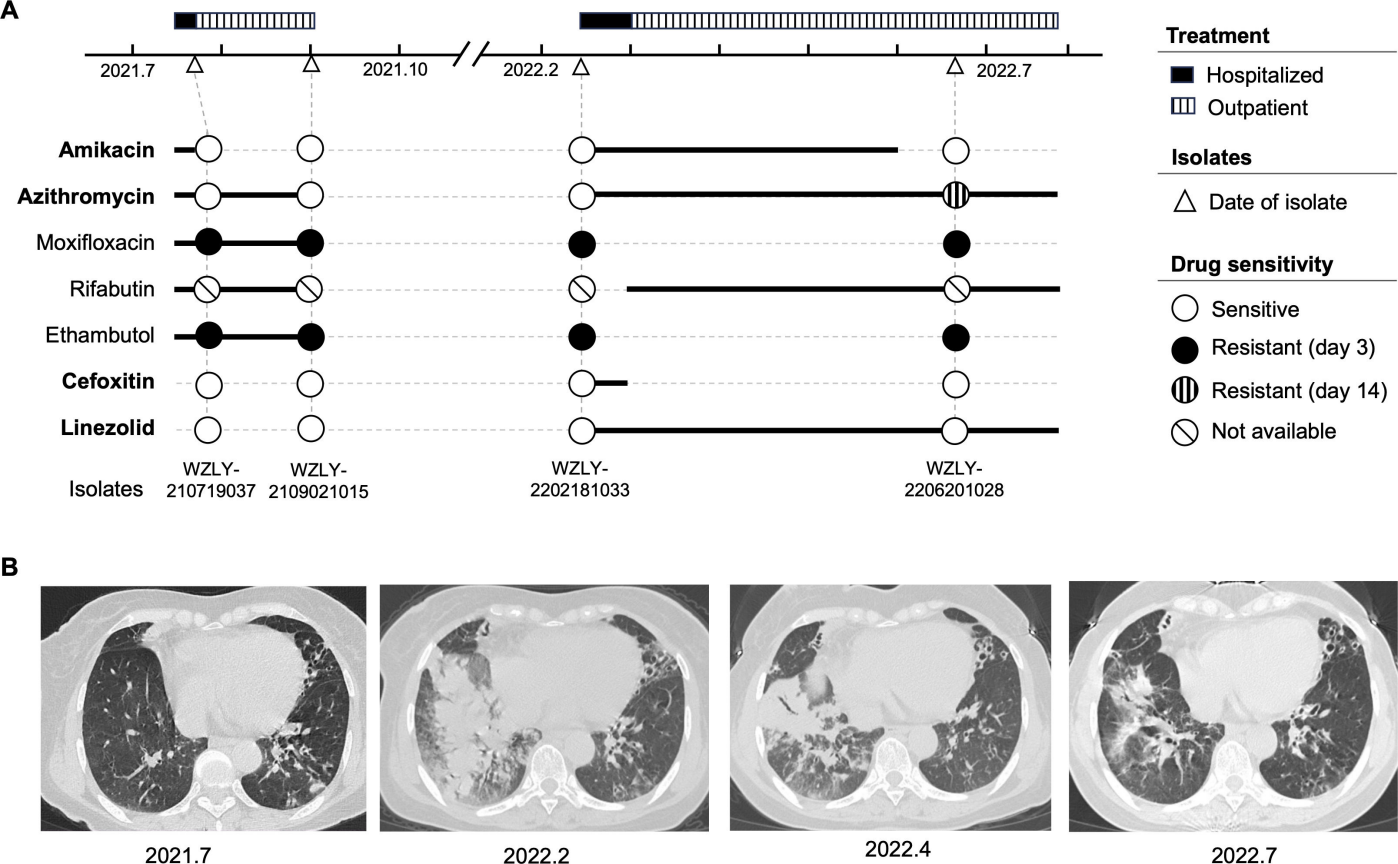
The emergence of inducible macrolide resistance during treatment. (**A**) Medication regimens during treatment (including both inpatient and outpatient cares), bacterial strain isolation, and drug susceptibility testing results. (**B**) Changes in chest CT scans of the patients at different treatment stages.

A 62-year-old female patient with a medical history of bronchiectasis and GOLD stage II COPD was admitted to the hospital on 13 July 2021, presenting with fever, productive cough, and left lower-lobe infiltrates on chest computed tomography (CT) (Fig. 1A). The acid-fast bacilli smear of the sputum was positive (2+), but Xpert MTB/RIF testing was negative, suggesting NTM pulmonary disease. Empirical therapy with rifabutin, ethambutol, azithromycin, moxifloxacin, and amikacin was initiated. The patient’s symptoms improved, and she was discharged on 20 July. She continued outpatient treatment with azithromycin, rifabutin, ethambutol, and moxifloxacin. However, she discontinued therapy within one month due to adverse effects. In February 2022, the patient was readmitted with relapsed symptoms and fever (39°C). Chest CT showed multiple patchy areas of increased density in the right lower lobe (Fig. 1B). Because both sputum specimens collected in July and September 2021 were culture-positive for *M. abscessus*, targeted therapy was initiated with amikacin (0.4 g IV once daily), cefoxitin (2.0 g IV every 8 h), azithromycin (0.5 g orally once daily), and linezolid (0.6 g orally once daily), leading to clinical improvement and discharge on 2 March 2022. Outpatient therapy was continued with intramuscular amikacin (0.4 g IV daily), azithromycin (0.5 g orally once daily), linezolid (0.6 g orally once daily), and rifabutin (0.3 g orally once daily). The dosage of amikacin was reduced because the patient could not tolerate the adverse effects of the higher dose (0.6 g IV once daily) typically administered. Amikacin was discontinued after three months due to intolerance. Follow-up CT scans in April and July showed progressive radiological improvement (Fig. 1B). The patient was briefly hospitalized in late July for supportive care, after which therapy was discontinued on 27 July 2022 because of improving imaging and poor drug tolerance. At follow-up in March 2024, the patient reported only occasional cough without further symptoms.

Four isolates were cultured from sputum specimens collected during therapy. The three early isolates were susceptible to macrolides, consistent with an *erm*(41) C28 allele. By contrast, the final isolate displayed inducible macrolide resistance (Fig. 1A; Table S1), corresponding to an *erm*(41) T28 allele. This raised two possibilities: (i) reinfection or mixed infection with a T28 strain, or (ii) *in vivo* mutation from C28 to T28. To discriminate between these scenarios, whole-genome sequencing was performed on all four isolates, and the sequencing data have been deposited in the NCBI database under accession number PRJNA1328661. Genomic variants were identified using both assembly-based and mapping-based pipelines, with GCF_020735345.1 as the reference genome. In the assembly-based pipeline, draft genomes were assembled using Shovill (v1.0.9), aligned to the reference with Minimap2 (v2.26), and variants were identified with Paftools. In the mapping-based pipeline, read alignment and variant calling were performed using Snippy (v4.6.0). A reference set of 233 diverse *M. abscessus* genomes, selected from 1,224 available genomes in the NCBI genome database with pairwise genomic distances >100 SNPs, was included. Whole genome alignment and phylogenetic analysis were performed according to our previous study (16).

**Fig 2:**
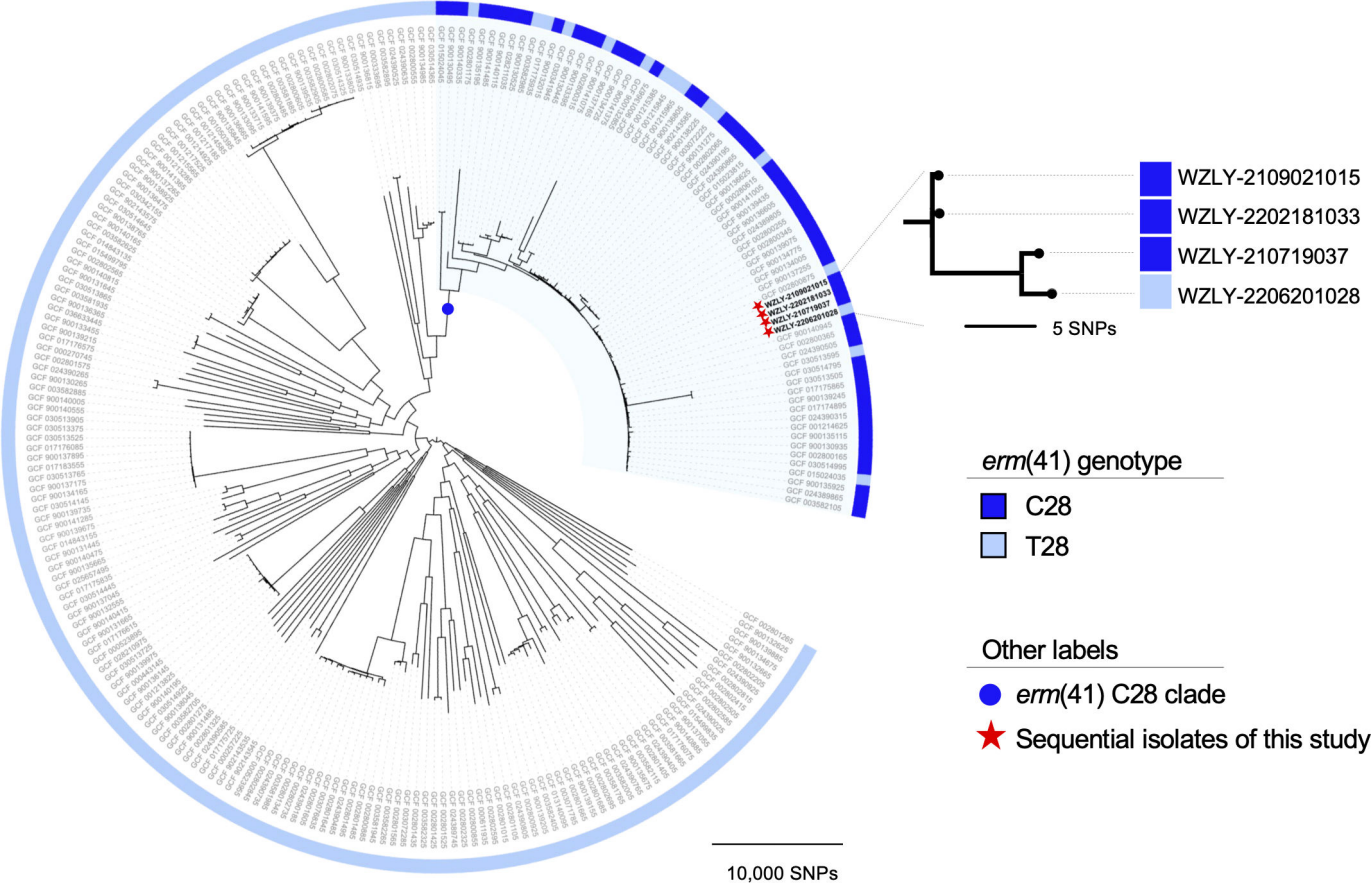
The maximum likelihood phylogeny based on the whole-genome alignment of four sequential isolates in this study together with 233 *M*. *abscesses* strains representative of global diversity.

According to the phylogeny, all *erm*(41) C28-type strains in the reference set formed a monophyletic clade (the C28 lineage; Fig. 2), consistent with a single ancestral origin (17). The four isolates of this study clustered tightly, differing by 0–9 SNPs, indicating they derived from the same infecting strain. A total of 12 genomic variants (10 SNPs and 2 indels; Table S2) were consistently detected by both pipelines across the four isolates, and no evidence of recombination was detected. The final isolate carried an *erm*(41) T28 allele, whereas the earlier three carried C28, confirming an *in vivo* C28T mutation during treatment (Fig. 2). The T28 allele of the final isolate exhibited three SNPs relative to the classical T28 allele in ATCC 19977, but only one SNP relative to the classical C28 allele, further supporting acquisition of the C28T mutation via spontaneous mutation (Fig. S1).

Our findings provide the first clinical evidence of an *erm*(41) C28T mutation emerging during antimicrobial therapy, resulting in acquired inducible macrolide resistance. This selection of resistance may have been related to the suboptimal dosage of amikacin. Because azithromycin and amikacin are the most effective drugs for treating *M. abscessus* infections, insufficient amikacin dosing may have reduced the selective pressure exerted by this agent, leaving azithromycin as the dominant selective pressure and thereby facilitating the emergence of the *erm*(41) C28T mutation. The *in vivo* emergence of this mutation during therapy highlights that inducible macrolide resistance can arise under drug-selective pressure. Consequently, in addition to screening for *rrl* mutations conferring constitutive macrolide resistance, clinicians should also consider the risk of *erm*(41) mutations leading to inducible resistance in strains that initially carry the C28 allele.

## Data Availability

The sequencing data of the four serial isolates have been deposited in the NCBI Sequence Read Archive (SRA) under BioProject accession number PRJNA1328661.

## SUPPLEMENTAL MATERIAL

Fig. S1 aac.01153-25-s0001.pdf Nucleotide sequence alignment of *erm*(41) alleles.

Table S1 aac.01153-25-s0002.docx MICs of clarithromycin for the clinical isolates.

Table S2 aac.01153-25-s0003.xlsx Genomic variants detected among the four clinical isolates.

ASM does not own the copyrights to Supplemental Material that may be linked to, or accessed through, an article. The authors have granted ASM a non-exclusive, world-wide license to publish the Supplemental Material files. Please contact the corresponding author directly for reuse.
