# Supplementary material for: Clinical emergence of inducible macrolide resistance mediated by acquired erm(41) C28T mutation in Mycobacterium abscessus
Source: Antimicrob Agents Chemother. 2025 Nov 6;69(12):e01153-25. doi: 10.1128/aac.01153-25 (PMC12691619; doi:10.1128/aac.01153-25)
Supplement: Fig. S1 — Nucleotide sequence alignment of erm(41) alleles. [file aac.01153-25-s0001.pdf]

Supplementary Figure 1. Nucleotide sequence alignment of *erm*(41) alleles, including ATCC 19977 (classical T28-type allele), GCF_020735345 (C28-type allele), and two clinical isolates from this study. The C28T mutation is indicated in red.

```
                               ↓28
                    ----:----|----:----|----:----|----:----|----:----|----:----|----:----|----:----|----:----|----:----|
ATCC19977 - erm(41) GTGTCCGGCCAACGGTCGCGACGCCAGTGGGGCTGGTATCCGCTCACTGATGACTGGGCGGCGCGGATCGTCGCCGAATCCGGTGTTCGCTCAGGGGAGT  100
GCF_020735345       ...........................C........................................................................  100
WZLY-210719037      ...........................C........................................................................  100
WZLY-2206202028     ...........................T........................................................................  100

                                                                      ↓159
                    ----:----|----:----|----:----|----:----|----:----|----:----|----:----|----:----|----:----|----:----|
ATCC19977 - erm(41) TCGTTGTGGATCTGGGCGCAGGACACGGCGCGCTGACGGCACATCTGGTTGCCGCTGGTGCCAGGGTGCTAGCCGTCGAGCTGCATCCGGGGCGGGCTCG  200
GCF_020735345       ..........................................................C.........................................  200
WZLY-210719037      ..........................................................C.........................................  200
WZLY-2206202028     ..........................................................C.........................................  200

                                                     ↓238
                    ----:----|----:----|----:----|----:----|----:----|----:----|----:----|----:----|----:----|----:----|
ATCC19977 - erm(41) ACACCTTCGTTCACGGTTTGCCGAGGAAGATGTCCGGATAGCGGAAGCGGACCTGCTCGCCTTCCGGTGGCCGCGACGGCCATTTCGGGTGGTGGCGAGC  300
GCF_020735345       .....................................G..............................................................  300
WZLY-210719037      .....................................G..............................................................  300
WZLY-2206202028     .....................................G..............................................................  300

                                         ↓330
                    ----:----|----:----|----:----|----:----|----:----|----:----|----:----|----:----|----:----|----:----|
ATCC19977 - erm(41) CCGCCCTACCAAGTCACCAGCGCACTGATACGGAGTCTCTTGACGCCGGAATCCCGGCTGCTGGCTGCCGACCTGGTGCTGCAGCGCGGGGCTGTGCACA  400
GCF_020735345       .............................C......................................................................  400
WZLY-210719037      .............................C......................................................................  400
WZLY-2206202028     .............................C......................................................................  400

                    ----:----|----:----|----:----|----:----|----:----|----:----|----:----|----:----|----:----|----:----|
ATCC19977 - erm(41) AACATGCGAAGCGAGCACCTGTTCGCCATTGGACGCTACGGGCCGGAATCACATTGCCGCGAAGCGCTTTCCATCATCCACCGCAGGTGGATTCGTCGGT  500
GCF_020735345       ....................................................................................................  500
WZLY-210719037      ....................................................................................................  500
WZLY-2206202028     ....................................................................................................  500

                    ----:----|----:----|----:----|----:----|----:----|----:----|----:----|----:----|----:----|----:----|
ATCC19977 - erm(41) GCTGGTGATCAGGCGGCGCTGA                                                                                522
GCF_020735345       ......................                                                                                522
WZLY-210719037      ......................                                                                                522
WZLY-2206202028     ......................                                                                                522
```
