# Supplementary material for: Clinical emergence of inducible macrolide resistance mediated by acquired erm(41) C28T mutation in Mycobacterium abscessus
Source: Antimicrob Agents Chemother. 2025 Nov 6;69(12):e01153-25. doi: 10.1128/aac.01153-25 (PMC12691619; doi:10.1128/aac.01153-25)
Supplement: Table S1 — MICs of clarithromycin for the clinical isolates. [file aac.01153-25-s0002.docx]

**Supplementary Table 1**. Minimum inhibitory concentrations (MICs) of clarithromycin for clinical isolates at different incubation times.

| Time | Clarithromycin MIC (μg/mL) | | | |
| --- | --- | --- | --- | --- |
|  | WZLY-210719037 | WZLY-2109021015 | WZLY-220218033 | WZLY-2206201028 |
| day 3 | ≤0.06 | 0.25 | ≤0.06 | 0.5 |
| day 4 | ≤0.06 | 0.25 | ≤0.06 | 0.5 |
| day 5 | 0.12 | 0.25 | ≤0.06 | 1 |
| day 7 | 0.12 | NA | 0.12 | 4 |
| day 10 | 0.25 | NA | 0.12 | 8 |
| day 14 | 0.25 | 0.5 | 0.25 | >16 |

Note: MICs were determined by broth microdilution according to CLSI guidelines. NA, not available.
